# Predicting clinically significant motor function improvement after contemporary task-oriented interventions using machine learning approaches

**DOI:** 10.1186/s12984-020-00758-3

**Published:** 2020-09-29

**Authors:** Hiren Kumar Thakkar, Wan-wen Liao, Ching-yi Wu, Yu-Wei Hsieh, Tsong-Hai Lee

**Affiliations:** 1 Department of Computer Science Engineering and School of Engineering and Applied Sciences, Bennett University, Plot Nos 8-11, TechZone II, Greater Noida, 201310 Uttar Pradesh India; 2 Department of Occupational Therapy and Graduate Institute of Behavioral Sciences, College of Medicine, Chang Gung University, No. 259, Wenhua 1st Rd., Taoyuan, Taiwan; 3 Healthy Aging Research Center, Chang Gung University, Taoyuan, Taiwan; 4 Department of Physical Medicine and Rehabilitation, Linkou Chang Gung Memorial Hospital, Taoyuan, Taiwan; 5 Department of Neurology, Linkou Chang Gung Memorial Hospital, Taoyuan, Taiwan; 6 College of Medicine, Chang Gung University, Taoyuan, Taiwan

**Keywords:** Machine learning, Prognosis, Rehabilitation, Stroke, Motor function

## Abstract

**Background:**

Accurate prediction of motor recovery after stroke is critical for treatment decisions and planning. Machine learning has been proposed to be a promising technique for outcome prediction because of its high accuracy and ability to process large volumes of data. It has been used to predict acute stroke recovery; however, whether machine learning would be effective for predicting rehabilitation outcomes in chronic stroke patients for common contemporary task-oriented interventions remains largely unexplored. This study aimed to determine the accuracy and performance of machine learning to predict clinically significant motor function improvements after contemporary task-oriented intervention in chronic stroke patients and identify important predictors for building machine learning prediction models.

**Methods:**

This study was a secondary analysis of data using two common machine learning approaches, which were the k-nearest neighbor (KNN) and artificial neural network (ANN). Chronic stroke patients (N = 239) that received 30 h of task-oriented training including the constraint-induced movement therapy, bilateral arm training, robot-assisted therapy and mirror therapy were included. The Fugl-Meyer assessment scale (FMA) was the main outcome. Potential predictors include age, gender, side of lesion, time since stroke, baseline functional status, motor function and quality of life. We divided the data set into a training set and a test set and used the cross-validation procedure to construct machine learning models based on the training set. After the models were built, we used the test data set to evaluate the accuracy and prediction performance of the models.

**Results:**

Three important predictors were identified, which were time since stroke, baseline functional independence measure (FIM) and baseline FMA scores. Models for predicting motor function improvements were accurate. The prediction accuracy of the KNN model was 85.42% and area under the receiver operating characteristic curve (AUC-ROC) was 0.89. The prediction accuracy of the ANN model was 81.25% and the AUC-ROC was 0.77.

**Conclusions:**

Incorporating machine learning into clinical outcome prediction using three key predictors including time since stroke, baseline functional and motor ability may help clinicians/therapists to identify patients that are most likely to benefit from contemporary task-oriented interventions. The KNN and ANN models may be potentially useful for predicting clinically significant motor recovery in chronic stroke.

## Introduction

Stroke is one of the leading causes of long-term disability [1]. Most stroke patients suffer from upper limb hemiparesis that significantly impairs their functional abilities and quality of life [2]. To help patients restore function, healthcare professionals have to provide rehabilitation interventions that are effective for each patient based on predicted outcomes. Nevertheless, making accurate prediction remains to be a challenging task due to the heterogeneous characteristics and recovery patterns among stroke patients [3].

With the recent advancement in technology, new techniques have been developed to assist clinicians/therapists in predicting patient recovery. One promising new technique is machine learning. Machine learning utilizes computerized algorithms to optimize prediction. It has several advantages including the ability to process large volumes of data, detection of complex interactions between multiple variables and easy incorporation of new attributes/data into models [4]. These advantages make machine learning an ideal tool for processing complex healthcare informatics data to develop prediction models [5].

In stroke, machine learning techniques have been used for predicting motor and functional recovery in acute/subacute stroke patients. For example, Lin et al. evaluated whether machine learning models could predict recovery of activities of daily living in acute stroke patients [6]. Other studies assessed whether machine learning models could predict motor and/or cognitive improvement in acute/subacute stroke patients [7–9]. Results of these studies were promising with moderate to high accuracy; however, these studies primarily involved inpatient rehabilitation in acute/subacute stroke. Whether the machine learning methods can predict responses of stroke patients to outpatient rehabilitation interventions, such as contemporary task-oriented interventions at chronic stage of stroke remain unknown.

Contemporary task-oriented rehabilitation interventions including the constraint-induced movement therapy (CIMT), bilateral arm training (BAT), robot-assisted therapy (RT) and mirror therapy (MT) are commonly used to address motor dysfunction in chronic stroke patients [10]. Systematic reviews and meta-analysis studies showed that these contemporary interventions were effective in improving motor function in chronic stroke patients, and should be considered in clinical application [11–14]. Machine learning may be a useful tool to predict motor function improvement after contemporary task-oriented interventions, which may help to identify the responders to these interventions and facilitate practical use.

The purpose of this study was to determine the accuracy and performance of machine learning in predicting clinically significant motor function improvement after contemporary task-oriented interventions in chronic stroke patients and identify important predictors for building machine learning prediction models.

## Methods

### Study design

This was an observational cohort study that used secondary analysis of data from our randomized controlled trials [15–20]. Data screening were done by three investigators (Thakkar HK, Liao WW, and Hsieh YW). The three investigators first determined the eligibility of the data. Then, two investigators (Thakkar HK and Liao WW) checked the completeness of the patient data. Patients whom had completed interventions and assessments were included for analysis in this study.

### Participants

Two-hundred and thirty-nine chronic stroke patients were included. They were recruited from 4 hospitals in the northern part of Taiwan. Participants were screened by the trained occupational therapists in each hospital to determine the eligibility. Participants received interventions in the rehabilitation clinic of the hospital where they were originally recruited from Table 1 outlines the baseline characteristics of participants. The mean age of participants was 54.72 ± 11.12 years and 73% of the participants were men. The selection criteria were (1) a first-ever unilateral ischemic or hemorrhagic stroke, (2) more than 6 months post stroke, (3) baseline Fugl-Meyer assessment scale (FMA) scores between 18 and 60, indicating moderate to mild hemiparesis [21], (4) ability to follow study procedures (Mini-Mental State Examination $\ge$ 22), and (5) no concomitant neurological disorders such as dementia. The institutional review boards of participating hospitals approved the trials and all participants provided informed consents.

**Table 1:** Demographics and clinical characteristics of participants

Baseline variables	Participants (N = 239)
Age (years)	54.72 ± 11.12
Gender (male/female)	176/63
Side of lesion (right/left)	108/131
Time since stroke (months)	17.95 ± 13.55
FMA	40.68 ± 12.29
NHISS	2.92 ± 2.51
Brunnstrom stage proximal	4.05 ± 0.81
Brunnstrom stage distal	3.99 ± 1.04
MAL AOU	0.82 ± 0.84
MAL QOM	0.86 ± 0.9
FIM	114.18 ± 11.39
SIS mean scores	48.95 ± 18.29
SIS recovery scores (%)	64.15 ± 11.78

Value is mean ± standard deviation

*FMA* Fugl-Meyer Assessment Scale of Upper Extremity, *NIHSS* National Institutes of Health Stroke Scale, *MAL AOU* motor activity log-amount of use, *MAL QOM* motor activity log-quality of movement, *FIM* functional independence measure, *SIS* stroke impact scale

### Contemporary task-oriented interventions

All participants received interventions for 1.5 to 2 h per day with a total of 30 h of training across 3 to 4 weeks. The frequency and duration of training hours were similar to most contemporary task-oriented interventions studies [22–25]. Among these participants, 68 received CIMT, 29 received BAT, 77 received RT and 65 received MT. Certified occupational therapists that were carefully trained by the senior occupational therapists and the principal investigator (Wu CY) delivered these interventions.

For the CIMT intervention, participants practiced functional tasks with their paretic arms while the non-paretic arms were restrained with a mitt. The functional tasks were designed according to common daily living tasks. Participants’ non-paretic arms were additionally restrained for another 5 to 6 h outside of training hours in their homes [16, 17]. For the BAT, participants practiced bilateral movements using both paretic and non-paretic arms simultaneously in the symmetrical or alternating fashion during functional tasks [16, 17]. For the RT, participants practiced forearm supination/pronation and wrist flexion/extension using the Bi-Manu-Track robot system. Participants practiced training modes of passive, active and resistance modes in each session [19, 20]. For the MT, a mirror was placed in participants’ midsagittal plane between the arms. Participants could only see the non-paretic arm and its mirror reflection. Participants were required to look at the mirror and imagined that the mirror reflection of the non-paretic arm was the paretic arm while performing bilateral movements as simultaneously as possible [18]. For the RT and MT, the participants performed additional 15–30 min of functional task training in each session.

Participants were assessed within one week before and after interventions by the evaluators who were blinded to the study purpose and allocation of treatment interventions of participants.

### Classification of motor function improvement

The FMA was selected as the major outcome for classification of motor function improvement because it is a widely used outcome measure evaluating upper extremity motor function post stroke [26, 27]. The reliability and validity of FMA have been established in chronic stroke patients [26]. In this study, the minimal clinical important difference (MCID) of FMA (i.e., FMA changed scores = 4) was used as the criterion value to classify participants into high and low responders [28]. Participants with FMA changed scores greater than or equal to 4 were classified as high responders and participants with FMA changed scores less than 4 were classified as low responders. We selected the MCID as the threshold for binary classification because MCID is regarded as the meaningful clinical improvement which patients perceive as beneficial in everyday life after receiving interventions [28]. Using the MCID for building prediction models would be relevant and helpful for determining high and low responders in clinical practice.

### Candidate predictors

We selected thirteen potential predictors based on the literatures and the International Classification of Functioning, Disability and Health (ICF) framework to include the “body function and structure level” (e.g., the impairment level), as well as the “activity and participation level” (e.g., functional ability, activities of daily living function and quality of life) variables [3, 29, 30].These 13 variables were (1) personal characteristic attributes including age, gender, side of lesion and time since stroke, (2) baseline motor and functional ability attributes including FMA scores [27], National Institutes of Health Stroke Scale (NIHSS) scores [31], Brunnstrom stage of the proximal and distal arm [32], Motor Activity Log (MAL) amount of use (AOU) and quality of movement (QOM) scores [33], and Functional Independence Measure (FIM) scores [34], and (3) baseline quality of life attributes including the Stroke Impact Scale (SIS) baseline mean scores and recovery scores [35]. These variables are commonly used for representing recovery of stroke patients in research and clinical settings [3, 29, 30].

### Machine learning algorithms

Two machine learning algorithms, which were the k-nearest neighbor (KNN) and the artificial neural network (ANN), were used for developing prediction models. The KNN algorithm is one of the most extensively used data mining tool to classify and predict patterns of health informatics data [36, 37]. The KNN algorithm predicts that similar objects would exist in close proximity; as a result, it labels the class of the target based on its surrounding k neighbors [38]. The KNN algorithm calculates the Euclidean distance between the target and its neighbors, and finds k neighbors that are closest to the target. It then determines the class of the target based on the majority of classes of these k neighbors. For example, the participant will be predicted to be a high responder if the majority of his/her neighbors are high responders. This prediction method is similar to the clinical decision making process made by clinicians/therapists. In most cases, a clinician/therapist may be likely to recommend a particular intervention to a new patient if the profile of this new patient matches the profiles of those patients that were successfully treated by this particular intervention. The KNN algorithm can thus be thought of as an artificial expert system that predicts responses of participants based on extensive experience gained from training [36].

The ANN algorithm is inspired by the biological neural networks of the human brain [39]. Similar to the human neural network, the ANN computing system consists of several neurons/nodes in different layers including the input, hidden and output layers. Neurons in these layers are interconnected with each other, and the links between the neurons can be enforcing or inhibitory. The input layer contains the data that entered into the ANN algorithms. The hidden layers are in between the input and output layers and are subsequent products of computations between each layer. The hidden layers take weighted inputs from the input layer, perform computations and produce a net input which is then applied with activation functions to generate the final output/classification result to the output layer. The output layer receives connections from the hidden layer and returns the prediction value of the output variables. The advantage of the ANN algorithms is that it can capture complicated non-linear relationship between the input and output variables through computations in the hidden layers, which makes it one of the ideal tools for outcome prediction in stroke patients [40]. The feedforward back propagation ANN algorithm was used in this study [41, 42]. Based on the ML literatures, we adopted one hidden layer and determined the optimal numbers of hidden neurons in the hidden layer using the k-fold cross validation method [40–42].

### Feature selection procedure

The feature selection procedure was adopted to reduce the unnecessary attributes and identify important ones contributing the prediction accuracy [43]. A popular machine learning-based feature selection method, which was the information gain ratio method, was employed [9, 44–46]. This feature selection method examined the influence (i.e., information gain ratio) of each attribute to the output classification (i.e., FMA classes) using the ranker search method [44–46]. A higher gain ratio indicates a greater contribution of this attribute to the output classification [46]. Attributes with higher gain ratio were used for development of the KNN and ANN models. In addition, the KNN and ANN models with all 13 attributes were also constructed to demonstrate the differences of prediction performance between models with 13 attributes and models with key attributes identified by the feature selection method.

### Model development and testing

**Fig. 1:**
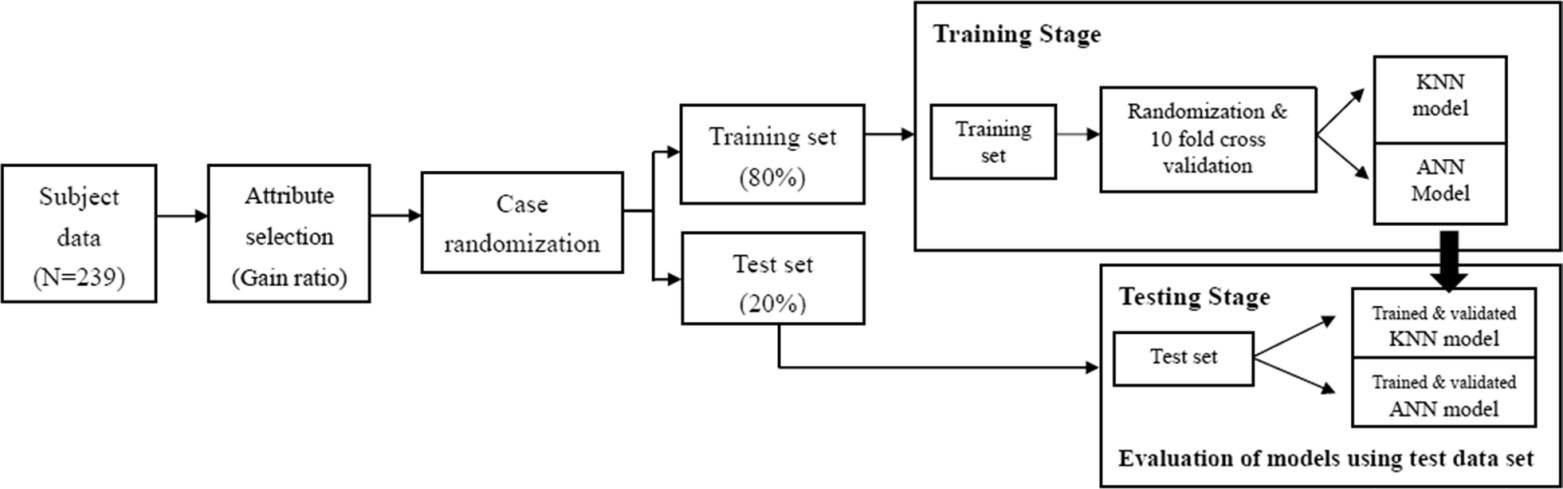
The flow chart of model development and validation process

Figure 1 illustrates the model development and testing process. To develop KNN and ANN models, data were randomized and divided into a training data set (80% data) and a test data set (20% data) [42]. The training data set was used for developing the models and the test data set was used for final examination of model performance. The tenfold cross validation procedure was used to train and tune the models [47]. During the tenfold cross validation process, the training data were split into 10 groups with 9 groups of data used for training the model while the remaining one group used for validating the model. This process was repeated until all groups of data had been trained and validated. The tenfold cross validation process was also used for tuning the hyper-parameters of the KNN (the k value) and ANN (the numbers of neurons in the hidden layer) models [7]. The numbers of k examined ranged from 1 to 10 and the hidden neurons examined were 2, 3, 4, 5 and 6. These values were selected based on suggestions from the KNN and ANN literatures [7, 36–42]. We found that k = 3 (KNN model) and the hidden neurons = 4 in one hidden layer (ANN model) provided the best prediction accuracy. As a result, these hyper-parameters were used for building models. After the models were built, the test data set was entered into the models to evaluate model performance.

### Model performance metrics

The performance of KNN and ANN models was evaluated using standard performance metrics including (1) accuracy, (2) recall, (3) specificity, (4) precision, (5) negative predictive value (NPV), (6) F1 scores and (7) area under the receiver operating characteristic curve (AUC-ROC) [48]. Accuracy is an overall index that considers true positive (TP), true negative (TN), false positive (FP) and false negative (FN)) together. Accuracy was computed as the sum of TP and TN divided by the sum of all 4 classes. Recall (i.e., sensitivity) is the ratio of participants that are correctly identified by our models as positive to those whom are positive in reality. Recall is calculated as TP divided by the sum of TP and FN. Specificity is the ratio of participants that are correctly identified by our models as negative to whom are negative in reality. Specificity was calculated as TN divided by the sum of TN and FP. Precision (also called positive predictive value, PPV) is the ratio of correctly identified as positive by our model to those who are labeled to the positive class by our model. Precision was calculated as TP divided by the sum of TP and FP. Negative predictive value (NPV) is the ratio of correctly identified as negative by our model to those who are labeled to the negative class by our model. NPV was calculated as TN divided by the sum of TN and FN. F1 scores are the harmonic mean of precision and recall and is a combination index. The AUC-ROC was calculated as the ratio of the area under the ROC curve to the total area. The AUC-ROC represents the ability of the model to distinguish between classes.

### Statistical analysis

Categorical variables were coded and continuous variables were standardized. The Waikato Environment for Knowledge Analysis (Weka) 3.8.3 developed by the University of Waikato, New Zeeland was used for feature selection, model development and testing [49]. Weka has been extensively used for constructing machine learning models in various fields including healthcare and technology [9, 50–52].

## Results

Three most important attributes were identified by the feature selection procedure, which were time since stroke (gain ratio = 0.25), baseline FIM scores (gain ratio = 0.24) and baseline FMA scores (gain ratio = 0.15). The gain ratio for the other 10 attributes was 0. As a result, time since stroke, baseline FIM scores and baseline FMA scores were used for developing the KNN and ANN models.

The accuracy of KNN model with three attributes was 85.42%, precision (PPV) was 0.85, recall (sensitivity) was 0.85, specificity was 0.67, NPV was 0.8, the F1 scores were 0.84, and the AUC-ROC was 0.89. The accuracy of the ANN model with three attributes was 81.25%, precision (PPV) was 0.8, recall (sensitivity) was 0.81, specificity was 0.49, NPV was 0.8, the F1 scores were 0.8, and the AUC-ROC was 0.77. Table 2 summarizes the performance metrics of KNN and ANN models. The performance of KNN and ANN models with the three attributes was better than those with all 13 attributes (Table 2). Table 3 shows the confusion matrix of the test samples of the KNN and ANN models.

**Table 2:** Model performance metrics of KNN and ANN models with the 3 and 13 attributes

Models	Accuracy (%)	Recall (sensitivity)	Specificity	Precision (PPV)	NPV	F1 scores	AUC-ROC
3 attributes							
KNN	85.42	0.85	0.67	0.85	0.8	0.85	0.89
ANN	81.25	0.81	0.49	0.80	0.8	0.79	0.77
13 attributes							
KNN	60.42	0.6	0.37	0.62	0.36	0.61	0.48
ANN	68.75	0.69	0.51	0.7	0.49	0.69	0.71

*KNN* k-nearest neighbors, *ANN* artificial neural network, *PPV* positive predictive value, *NPV* negative predictive value, *AUC-ROC* area under the receiver operating characteristic curve

**Table 3:** Confusion matrix of the test samples (N = 48)

Confusion matrix (3 attributes) Test samples = 48; Actual low responders = 12; Actual high responders = 36		
	Predicted: low responders	Predicted: high responders
KNN		
Actual: low responders	7	5
Actual: high responders	2	34
ANN		
Actual: low responders	4	8
Actual: high responders	1	35

*KNN* k-nearest neighbors, *ANN* artificial neural network

## Discussion

Our results showed that machine learning algorithms can accurately predict motor function improvement in above 80% of the participants. The KNN model had 89% chance and the ANN model had 77% chance to distinguish between high and low responders. Furthermore, we identified three most important attributes, which were the time since stroke, baseline FIM scores and baseline FMA scores. The combination of these three attributes made better prediction than all attributes together. The sensitivity, PPV, NPV and F1 scores of the KNN and ANN models were good; however, the specificity was relatively low in the ANN model. The KNN model had overall better prediction performance than the ANN model.

Consistent with the findings of previous studies, our study showed that machine learning methods are feasible and applicable for predicting recovery of stroke patients [6–9]. Furthermore, we expand findings of previous studies by showing that machine learning methods could also make accurate prediction for post-intervention improvements of common task-oriented interventions in individuals with chronic stroke. The prediction performance of our models was comparable to those reported in the studies of acute/subacute stroke. For example, one previous study found a prediction accuracy of 83% using random forest models in acute stroke patients [9]. Another two studies found model discriminating ability between 77 and 89% using various types of machine learning methods (e.g. support vector machine and logistic regression) in acute stroke patients [6, 8]. Similarly, in the present study, we identified prediction accuracy of 85% and 81% and discriminating ability of 89% and 77% with KNN and ANN models. Although the prediction performance was similar between ours and previous studies, predicting changes in chronic stroke patients could be a much more difficult task because changes during the chronic period were not as evident as those in the acute/subacute period of stroke. Our study demonstrated that machine learning approaches were still capable of predicting functional changes in chronic stroke.

Three most important attributes were identified, which were time since stroke, baseline FIM scores and FMA scores. Time since stroke indicates the remaining levels of neural plasticity post stroke [53]. The remaining levels of neural plasticity may affect how the brain re-organizes itself and the resulting neurophysiological processes, such as cortical excitability and interhemispheric inhibition during the task-oriented interventions, which in turn will impact motor function improvement [53, 54]. Baseline FIM scores indicate the initial functional ability of the participants. Studies have showed that individuals’ FIM scores at admission could predict improvements at discharge and long term care requirement [55, 56]. Similarly, in the present study, we found that individuals’ FIM scores prior to the task-oriented training can determine post-intervention improvement. As a result, FIM may be a useful outcome to predict recovery in both acute and chronic stroke. Baseline FMA scores indicate initial motor function of the paretic arm. Several prediction model studies have found that baseline motor function was associated with recovery after stroke [57–59]. A recent study also found that motor recovery could be predicted by the initial FMA scores in 5 different subgroups of stroke patients [60]. Furthermore, contemporary task-oriented interventions emphasized repetitive practice of paretic arm movements to restore motor function. It is thus reasonable to find initial motor function crucial for post-intervention improvements.

These three attributes represent the baseline characteristics and impairment levels of participants, which may be difficult to modify. However, these three attributes could serve as useful indicators that help clinicians to identify chronic stroke patients who may benefit the most from the contemporary rehabilitation interventions. Subsequently, these interventions can be provided to the suitable patients in time. Based on our findings, we recommend clinicians/therapists to record the duration of time post stroke and assess at least the baseline FIM and FMA scores before applying contemporary task-oriented interventions in chronic stroke patients. The information provided by these three attributes can inform clinicians/therapists of the recovery potentials of a particular chronic stroke patient and whether he/she would have better chances to benefit from contemporary task-oriented interventions. Assessing and recording these three attributes, instead of all 13 attributes, may help to save the workload in clinical settings and improve clinical practice efficiency.

Our study demonstrated that the initial level of impairments (i.e., baseline FMA scores) could predict whether participants reached clinically significant improvements after contemporary rehabilitation interventions. This finding was consistent with the “Proportional recovery rule” identified in previous stroke prediction model studies [61–65]. The “Proportional recovery rule” is the idea that most stroke patients will recover approximately 70% to 80% of their potential based on the differences between the initial and the maximum FMA scores [61–65]. For example, Winters et al. found that about 70% of their study patients demonstrated a fixed proportional paretic arm recovery (i.e., 78%) from acute to chronic phase of stroke [63]. According to this model, the initial FMA scores play a critical role in predicting recovery potentials of stroke patients. However, the “Proportional recovery rule” has been criticized due to the mathematical coupling issue, where the initial FMA scores were part of the dependent (final FMA scores-initial FMA scores) as well as independent variables (maximum FMA scores-initial FMA scores) in a regression model [66, 67]. In this study, we adopted machine learning methods rather than regression analyses to construct prediction models and we found that initial FMA scores also critical for predicting stroke recovery. Our results along with the others indicate that the initial impairment level may need to be considered during stroke rehabilitation processes [61–65]. Future studies could adopt different types of machine learning algorithms such as support vector machine to examine whether the proportional recovery rule still holds true in different types of machine learning prediction models.

In addition, similar to the “Proportional recovery rule” studies, our machine learning models also showed that there might be non-fitters of the “Proportional recovery rule” and they could not be accurately predicted based on the initial impairment level [61–65]. This could be the reason why the accuracy of our machine learning predication models was around 80%. It may be possible that these non-fitters require more intensive training than the fitters to be able to trigger proportional recovery and benefit from rehabilitation interventions [68, 69]. Future study could adjust the intensity (e.g., duration and/or frequency) of contemporary rehabilitation interventions to examine if this would impact prediction accuracy.

In addition to the three clinical variables identified in this study, studies have found that other types of predictors were also relevant for predicting stroke recovery in acute, subacute and chronic stroke patients. These predictors included the kinematic variables (e.g., reaction time, movement speed and path ratio) and neurophysiological variables such as motor evoked potentials (MEP). For example, Stinear et al. found that the strength of the shoulder abduction and finger extension in combination with MEP could predict patients’ motor recovery at 3 months post stroke [70]. Majeed et al. found that kinematic variables such as the speed ratio and numbers of speed peaks contributed to prediction of changes of FMA scores after a three-week intervention in chronic stroke patients [71]. Future studies could include the three clinical predictors identified in this study (i.e., time since stroke, baseline FMA and FIM scores) as well as kinematic and neurophysiological variables in the ML prediction models to determine if inclusion of various types of variables would optimize prediction performance.

Given that no one algorithm works best for every problem, it is recommended to use multiple machine learning algorithms to examine data [4]. Following the recommendation, we adopted two common machine learning algorithms, which were the KNN and ANN. Both algorithms can process linear and non-linear relationship within the data and therefore suitable for building prediction models for complicated health informatics data [72]. We found that both models can predict responses of over 80% of participants and have approximately 80% chance or above to distinguish between high and low responders, indicating that the KNN and ANN algorithms may be suitable tools for predicting post-intervention changes in chronic stroke patients. However, the overall performance of KNN model was better than that of the ANN model. This result was consistent with the finding of two previous studies that examined the performance of KNN and ANN in classifying responses of brainwave/imaging data [73, 74]. Those studies also identified higher accuracy in the KNN than ANN models. In addition to the accuracy, the specificity was also lower in the ANN than the KNN models although other performance metrics (i.e., sensitivity, AUC-ROC, PPV and NPV) were comparable between these two models. This result was similar to the findings of one previous study that classified brain imaging data using the logistic regression and ANN model [75]. In that study, the specificity was also low in the ANN model. Two potential reasons may explain why the prediction performance (i.e., accuracy and specificity) of ANN model was weaker than that of the KNN model. First, the sample size of the data may not be optimal for constructing the ANN prediction model. Compared with the KNN, the ANN is a much more complex algorithm and usually requires larger data set [72, 76]. It is possible that inclusion of more participants may improve the prediction performance of the ANN model. Second, the low specificity of ANN model could be due to fewer numbers of participants in the low responder class in the test data set [72, 76, 77]. It is plausible that increasing numbers of patients in the low responder class may enhance the specificity of the ANN model. However, in the real world, it may be difficult to obtain a balanced dataset with equal numbers of patients in the low responder and high responder group because only those interventions/treatments that have been demonstrated to be beneficial for most patients will be regularly performed. As a result, the amounts of low responders are often smaller than those of high responders in clinical settings. On the other hand, our result suggests that the KNN algorithm may already be a potentially useful tool for outcome prediction in chronic stroke patients. The high sensitivity with moderate specificity as well as good predictive value and discriminating ability indicates that the KNN model could be considered in outcome prediction of stroke patients in future clinical application.

### Study limitations

Six limitations should be considered. First, our outcome prediction was focused on contemporary task-oriented interventions. Future studies could examine whether the identified features of this study could generalize to other types of interventions. Second, we examined predictions of motor function. Future study can explore if machine learning can accurately predict improvements in other domains (e.g., quality of life). Third, our predictions were based on the changes immediately after interventions. Future studies could explore whether machine learning methods can be used to predict retention in the follow-up period. This will help to identify patients that will have lasting improvements after task-oriented interventions. Fourth, there were fewer patients in the low responder than the high responder group, which may potentially affect the prediction performance (i.e., specificity) of the ANN model although other performance metrics, including accuracy, sensitivity, positive/negative predictive value and AUC-ROC were sufficient in the ANN model. Future study could include a larger sample of stroke patients with more low responders and examine if the specificity of the ANN model would improve. Fifth, we used the binary classification method to construct prediction models. Although the performance of our binary classification models was good, it is still possible that multi-level classification method may increase prediction accuracy. Future studies could divide patients into three groups (i.e., the low, medium and high responder group) and determine if the multi-level classification method would increase prediction accuracy in stroke patients. Sixth, we only examined prediction performance of the KNN and ANN algorithms. Future studies could include other types of machine learning algorithms such as decision tree or support vector machine and compare their performance with the KNN and ANN algorithms. This will help to identify the optimal ML algorithm for predicting motor recovery in chronic stroke patients.

## Conclusions

Machine learning-based approaches such as the KNN and ANN may accurately predict clinically significant motor function improvement after the contemporary task-oriented interventions in chronic stroke patients and therefore could be considered in clinical settings. We suggest including at least three predictors, which are time since stroke, initial FIM and FMA scores into the machine learning models to optimize prediction accuracy. Future studies with a different sample of chronic stroke patients and a larger sample size are warranted to validate the findings of this study.

## Data Availability

The datasets used and/or analyzed during the current study are available from the corresponding author on reasonable request.

## Abbreviations

ANN: artificial neural network
AUC-ROC: area under the receiver operating characteristic curve
BAT: bilateral arm training
CIMT: constraint-induced movement therapy
FIM: functional independence measure
FMA: Fugl-Meyer assessment scale
KNN: K-nearest neighbor
MAL AOU: motor activity log-amount of use
MAL QOM: motor activity log-quality of movement
MT: mirror therapy
NIHSS: National Institutes of Health Stroke Scale
RT: robot-assisted therapy
SIS: Stroke Impact Scale
